# Insulin signaling in skeletal muscle of HIV-infected patients in response to endurance and strength training

**DOI:** 10.1002/phy2.60

**Published:** 2013-08-22

**Authors:** Christa Broholm, Neha Mathur, Thine Hvid, Thomas Sahl Grøndahl, Christian Frøsig, Bente Klarlund Pedersen, Birgitte Lindegaard

**Affiliations:** 1 Department of Infectious Diseases, Centre of Inflammation and Metabolism, RigshospitaletCopenhagen, Denmark; 2 Section of Molecular Physiology, The August Krogh Centre, Department of Nutrition, Exercise and Sports, University of CopenhagenCopenhagen, Denmark

**Keywords:** HIV, insulin signaling, lipodystrophy, skeletal muscle, training

## Abstract

Human immunodeficiency virus (HIV)-infected patients with lipodystrophy have decreased insulin-stimulated glucose uptake. Both endurance and resistance training improve insulin-stimulated glucose uptake in skeletal muscle of HIV-infected patients, but the mechanisms are unknown. This study aims to identify the molecular pathways involved in the beneficial effects of training on insulin-stimulated glucose uptake in skeletal muscle of HIV-infected patients. Eighteen sedentary male HIV-infected patients underwent a 16 week supervised training intervention, either resistance or strength training. Euglycemic–hyperinsulinemic clamps with muscle biopsies were performed before and after the training interventions. Fifteen age- and body mass index (BMI)-matched HIV-negative men served as a sedentary baseline group. Phosphorylation and total protein expression of insulin signaling molecules as well as glycogen synthase (GS) activity were analyzed in skeletal muscle biopsies in relation to insulin stimulation before and after training. HIV-infected patients had reduced basal and insulin-stimulated GS activity (%fractional velocity, [FV]) as well as impaired insulin-stimulated Akt^thr308^ phosphorylation. Despite improving insulin-stimulated glucose uptake, neither endurance nor strength training changed the phosphorylation status of insulin signaling proteins or affected GS activity. However; endurance training markedly increased the total Akt protein expression, and both training modalities increased hexokinase II (HKII) protein. HIV-infected patients with lipodystrophy have decreased insulin-stimulated glucose uptake in skeletal muscle and defects in insulin-stimulated phosphorylation of Akt^thr308^. Endurance and strength training increase insulin-stimulated glucose uptake in these patients, and the muscular training adaptation is associated with improved capacity for phosphorylation of glucose by HKII, rather than changes in markers of insulin signaling to glucose uptake or glycogen synthesis.

## Introduction

Human immunodeficiency virus (HIV)-infected patients are treated with highly active combination antiretroviral therapy (HAART), which as a side effect leads to development of lipodystrophy. HIV-associated lipodystrophy is characterized by a loss of subcutaneous fat and relative gain in visceral fat leading to severe metabolic disturbances like insulin resistance (Grinspoon and Carr 2005).

Patients with HIV-associated lipodystrophy show both impaired insulin-stimulated whole-body oxidative glucose disposal (Behrens et al. 2002) and impaired nonoxidative glucose disposal (van der Valk et al. 2001; Behrens et al. 2002; Andersen et al. 2003; Haugaard et al. 2005) indicating peripheral insulin resistance. Defective oxidative glucose disposal in HIV-infected patients is caused by impaired glucose transport and phosphorylation (Behrens et al. 2002), suggesting defects in glucose phosphorylating enzymes like hexokinase II (HKII). In both normal and diabetic subjects, storage of glucose as muscle glycogen accounts for the majority of the nonoxidative glucose disposal and for most of the total body glucose uptake during insulin infusion (Shulman et al. 1990). Therefore, the reduced nonoxidative glucose disposal in patients with HIV-associated lipodystrophy is believed to reflect impaired glycogen synthesis (Andersen et al. 2003; Haugaard et al. 2005), and molecular defects at the level of glycogen synthase (GS), glycogen synthase kinase (GSK)-3, and Akt have been demonstrated in skeletal muscle of patients with HIV-associated lipodystrophy (Haugaard et al. 2005).

It is well established that physical activity is beneficial for improving insulin sensitivity as individuals who maintain a physically active lifestyle are less likely to develop insulin resistance, impaired glucose tolerance, and type 2 diabetes (Hawley 2004; Pedersen 2006). Based on studies in healthy and diabetic subjects, the mechanisms underlying the beneficial effects of exercise on insulin sensitivity in muscle seem to be associated with changes in protein composition of skeletal muscle with relevance to the actions of insulin (e.g., glucose transporter-4 [GLUT4], HKII, insulin receptor [IR], Akt, TBC1D4 [previously called AS160], and GS) rather than major changes in insulin signaling capacity as determined by phosphorylation status or enzyme activities (Henriksen 2002; Zierath 2002; Holten et al. 2004; Frosig et al. 2007).

Only few studies have investigated the effect of training on insulin sensitivity in patients with HIV-associated lipodystrophy, and conflicting results exist (Jones et al. 2001; Smith et al. 2001; Yarasheski et al. 2001; Thoni et al. 2002; Driscoll et al. 2004; Dolan et al. 2006; Robinson et al. 2007). We recently performed a study investigating the effect of 16 weeks supervised endurance and resistance training on insulin sensitivity, measured by the euglycemic–hyperinsulinemic clamp technique with tracer infusion, in male HIV-infected patients with lipodystrophy. At baseline, HIV-infected patients had lower insulin sensitivity than healthy HIV-negative men, but both training modalities markedly increased peripheral insulin sensitivity (Lindegaard et al. 2008). Muscle biopsies were obtained during the euglycemic–hyperinsulinemic clamp performed in HIV-infected patients before and after both training interventions, and in the present study we aimed to define the possible intracellular pathways mediating the beneficial effects of endurance and resistance exercise on peripheral insulin sensitivity in HIV-infected patients with lipodystrophy.

## Research Design and Methods

### Participants

Thirty-nine HIV-infected men were recruited from the outpatient clinic of the Department of Infectious Diseases (Rigshospitalet, Copenhagen, Denmark). The detailed inclusion and exclusion criteria were previously described (Lindegaard et al. 2008). In short, the patients enrolled in the study were untrained according to Astrand et al. (1973), had received HAART treatment for at least 3 month prior to the study, and were defined as lipodystrophic. Of 39 patients recruited, 24 fulfilled the inclusion criteria, but four declined to participate and two withdrew from the training study because of severe back problems and psychiatric problems. Thus, 18 HIV-infected patients completed the study.

Fifteen age- and VO_2_max-matched HIV-negative healthy men served as controls for baseline measurement. Written and informed consent was obtained from all participants according to the requirements from the local ethical committee (KF 01-262/04) and the Helsinki Declaration II.

### Study protocol

All HIV-infected patients were randomized to either 16 weeks of endurance or strength training. Before, after 8 and after 16 weeks of training, HIV-infected patients performed a VO_2_max test on cycle ergometer and a three-repetition maximum (3-RM) strength test during six exercises: leg curl, pull-down, seated leg press, chest, press, seated rows, and leg extension. At baseline and after 16 weeks a euglycemic–hyperinsulinemic clamp with stable isotope infusion was performed as described previously (Lindegaard et al. 2008). Muscle biopsies were obtained before the clamp and 150 min after start of the clamp in musculus vastus lateralis using the Bergstøm biopsy needle technique (Bergstrom 1975). The participants were randomized to endurance training or strength training after the clamp procedure. All training sessions were supervised, and the subjects’ heart rate was continuously monitored. The subjects trained three times per week for 16 weeks. The endurance training protocol consisted of eight different programs with 35 min of interval training. The first 8 weeks, the mean intensity was targeted at 65% of VO_2_max and the last 8 weeks, it was 75% of VO_2_max. The strength training consisted of eight exercises (leg curl, pull-down, seated leg press, chest press, seated rows, leg extension, abdominal crunch, and back extension) in resistance training machines for 45–60 min. The resting intervals were 60–120 sec. The number of repetitions and sets changed every week. Compliance was noted at each training day, and if subjects missed a training day, a makeup was made.

### Muscle lysate preparation

Muscle tissue was freeze-dried and dissected free of visual blood, fat, and connective tissues. Depending on weight, muscle lysate was then prepared by the addition of 0.4–1.0 mL homogenization buffer (50 mmol/L Hepes, 10% glycerol, 20 mmol/L sodium pyrophosphate, 150 mmol/L sodium chloride, 1% NP-40, 20 mmol/L β-glycerophosphate, 10 mmol/L sodium fluoride, 2 mmol/L phenylmethanesulfonyl fluoride, 1 mmol/L ethylenediaminetetraacetic acid, 1 mM ethylene glycol tetraacetic acid, 10 μg/mL aprotinin, 10 μg/mL leupeptin, 2 mmol/L sodium orthovanadate, 3 mmol/L benzamidine) to the freeze-dried muscle tissue. The muscle tissue was then homogenized using cooled racks in a TissueLyser (Qiagen, Valencia, CA) for 1 min at 30 Hz followed by 15 min incubation on ice. The homogenization and incubation on ice were repeated two or three times depending on the degree of homogenization of the tissue. Homogenates were then rotated end over end for 1 h at 4°C and centrifuged at 16 000 *g* at 4°C for 25 min. The supernatant protein concentrations were determined with a Bio-Rad DC kit (Bio-Rad, Hercules, CA) using bovine serum albumin (BSA) as standard. All determinations were done in triplicate.

### Western blotting

Protein expression and protein phosphorylation were studied in muscle tissue homogenates by Sodium dodecyl sulfate-polyacrylamide gel electrophoresis using 4–12% Bis-Tris gels (Invitrogen, Taastrup, Denmark) and western blotting using polyvinylidene difluoride membranes (GE Healthcare, Little Chalfont, U.K.). The membranes were blocked for 1 h at room temperature in either 5% skim milk or 5% BSA and subsequently incubated overnight at 4°C with antibodies against AS160^Thr642^ (#4288; Cell Signaling, Danvers, MA), AS160 (# 07-741; Upstate, Millipore, Billerica, MA), Akt^thr308^ (#4056; Cell Signaling), Akt (#9272; Cell Signaling), GSK-3β (#9315; Cell Signaling), GSK-3β^ser9^(#9323; Cell signaling), HXKII (#6521; Santa Cruz, CA) and GS (#3893; Cell Signaling). For detection of GS site 3a+b (ser640 and ser644 cophosphorylation), an antibody was raised against the peptide PYPRPPASpVPPSpPSLSR as described (Hojlund et al. 2003). After overnight incubation, the membranes were incubated for 1 h at room temperature with a horseradish peroxidase-conjugated secondary antibody (DAKO, Glostrup, Denmark). Protein bands were detected using Supersignal West Femto chemiluminescence (Pierce, Rockford, IL) and quantified using a charge-coupled device image sensor (ChemiDocXRS; Bio-Rad) and software (Quantity One; Bio-Rad). To check for even loading and transfer, all membranes were stained with reactive brown (Sigma-Aldrich, St. Louis, MO).

### GS activity

GS activity was measured in duplicates in muscle homogenates by using a unifilter 350 microtiter plate assay (Whatman, Frisenette, Ebeltoft, Denmark) as described by Thomas et al. 1968.

### Statistics

**Figure 1:**
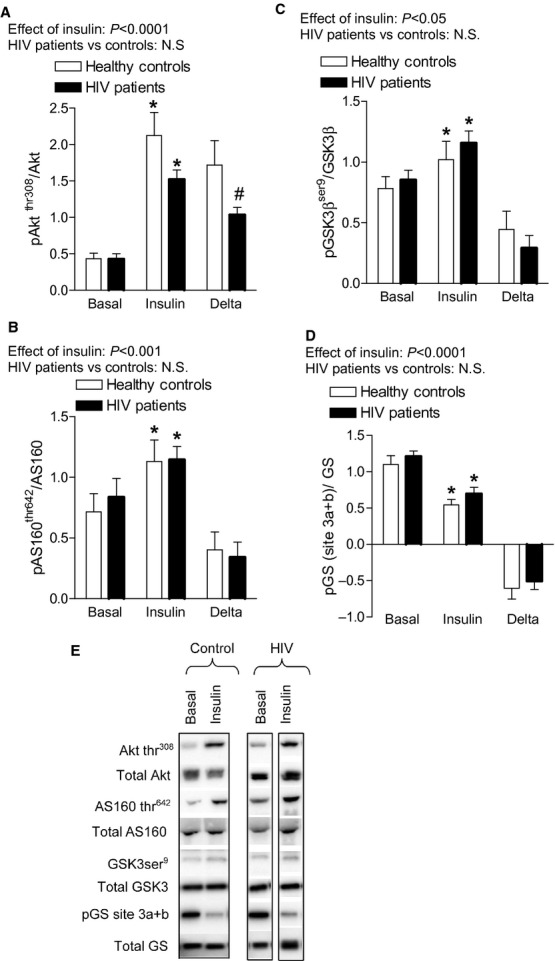
Phosphorylation level of insulin signaling molecules in skeletal muscle of 18 human immunodeficiency virus (HIV)-infected patients and 15 HIV-negative individuals at basal levels and after insulin infusion. (A) Phosphorylation of Akt^thr308^ related to total Akt protein expression, (B) Phosphorylation of AS160^thr642^ related to total AS160 protein expression, (C) Phosphorylation of GSK3β^ser9^ related to total GSK3β protein expression, (D) Phosphorylation of glycogen synthase GS at site 3a+b related to total GS protein expression, (E) Representative western blots are shown: Protein bands are from the same subject before and after insulin stimulation. The black line indicates that the samples were not loaded adjacent, but for each subject they were loaded on the same gel. Data are means ± SE. *P < 0.05 (Post hoc paired *t*-test comparing insulin values to basal values in the same group), #*P* < 0.05 unpaired *t*-test comparing changes (Δ) from basal to insulin stimulation between groups.

**Figure 2:**
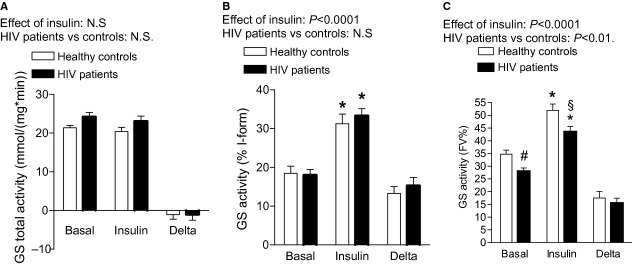
Activity measures of glycogen synthase (GS) in skeletal muscle of 18 HIV-infected patients and 15 HIV-negative individuals at basal levels and after insulin infusion. (A) Total GS activity, (B) GS activity given as percent I-form, (C) GS activity given as fractional velocity (FV) percent. Data are means ± SE. *P < 0.001 (post hoc paired *t*-test comparing insulin values to basal values in the same group), §*P* < 0.05 (post hoc *t*-test comparing insulin values between HIV-infected patients and controls), #*P* < 0.01 (post hoc *t*-test comparing basal values between HIV-infected patients and controls).

**Figure 3:**
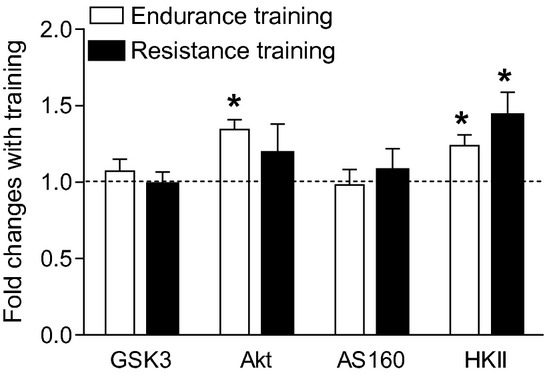
Protein content of GSK3, Akt, AS160, and HKII in skeletal muscle of HIV-infected patients before and after 16 weeks of endurance (*n* = 8) and strength training (*n* = 10). Values are shown as posttraining values divided by pretraining values thereby expressing fold changes in response to training. Dotted line shows pretraining levels. Values are means ± SE. *P < 0.05 (vs. pretraining levels).

**Figure 4:**
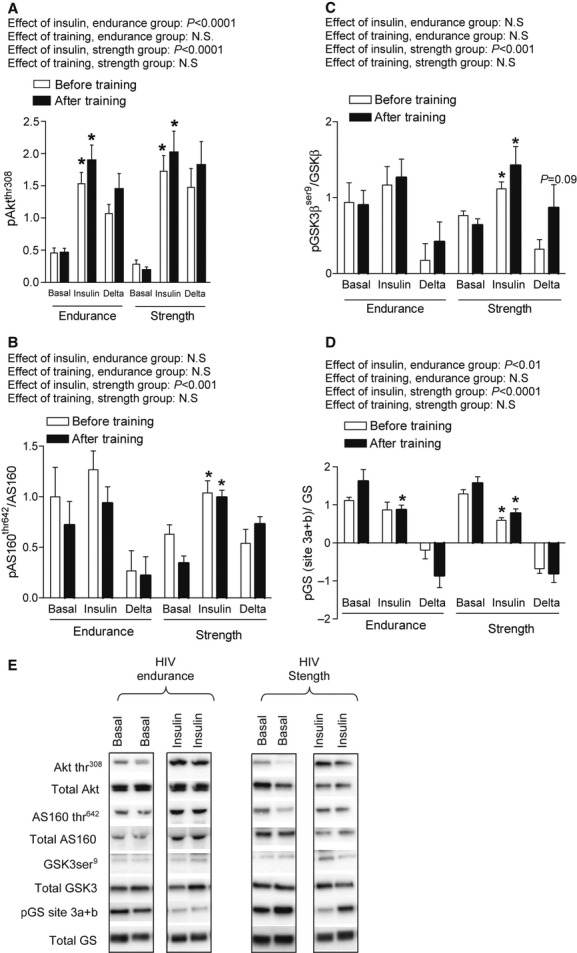
Phosphorylation level of insulin signaling molecules in skeletal muscle of HIV-infected patients before and after 16 weeks of endurance (*n* = 8) and strength training (*n* = 10) at basal levels and after insulin infusion. (A) Phosphorylation of Akt^thr308^, (B) Phosphorylation of AS160^thr642^ related to total AS160 protein expression, (C) Phosphorylation of GSK3β^ser9^ related to total GSK3β protein expression, (D) Phosphorylation of GS at site 3a+b related to total GS protein expression, (E) Representative western blot is shown: Protein bands are from the same subject before/after endurance and strength training, respectively. The black line indicates that the basal samples and insulin samples were not loaded adjacent, but for each subject they were loaded on the same gel. Data are means ± SE. *P < 0.05 (post hoc paired *t*-test comparing insulin values to basal values in the same group)

**Figure 5:**
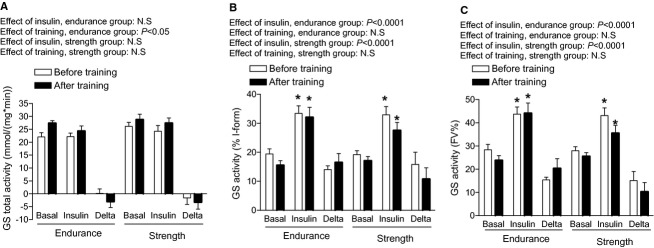
Activity measures of glycogen synthase (GS) in skeletal muscle of HIV-infected patients before and after 16 weeks of endurance (*n* = 8) and strength training (*n* = 10) at basal levels and after insulin infusion. (A) Total GS activity, (B) GS activity given as percent I-form, (C) GS activity given as fractional velocity (FV) percent. Data are means ± SE. *P < 0.05 (post hoc paired *t*-test comparing insulin values to basal values in the same group)

All analyses were performed using SAS software version 9.1.3. Data were evaluated using two-way analysis of variance (ANOVA) with repeated measures for effect of clamp and group (Figs. 1 and 2) or for effect of clamp and training (Figs. 4 and 5). The ANOVA analyses were performed separately for the endurance training and strength training groups. Student's *t*-test with bonferroni correction was used as post hoc test. The residuals obtained from the ANOVA models were evaluated, and the model was only accepted if the residuals were normally distributed. If data were not normally distributed, a logarithmic transformation was performed. Changes (Δ) from basal to insulin-stimulated values were compared between groups by unpaired *t*-test (Figs. 1, 2, and 4, 5). Changes in total protein expression before and after training were evaluated by paired *t*-test (Fig. 3). Data are presented as means ± SE unless otherwise indicated. A level of *P* < 0.05 was accepted as statistically significant.

## Results

The general adaptations to the present training regimen have been published previously (Lindegaard et al. 2008). In brief, HIV-infected patients were included on the basis of moderate lipoatrophy and were characterized by reduced limb fat mass, increased percentage of fat in the trunk, and an increased trunk-to-limb fat ratio. HIV-infected patients had increased blood plasma insulin concentrations, increased homeostatic model assessment for insulin resistance (HOMA-IR), and increased glucose and insulin area under the curves during an oral glucose tolerance test (OGTT) compared to controls (Table 1). During the euglycemic–hyperinsulinemic clamp, insulin-stimulated glucose uptake and changes (Δ) from basal to insulin-stimulated glucose uptake were lower in HIV-infected patients compared to controls (Table 1). Insulin-mediated glucose uptake was improved by both endurance training and strength training (Table 2). Only strength training increased total lean body mass (2.5 kg) and decreased total fat mass (3.3 kg).

**Table 1:** Baseline characteristics for HIV patients and healthy controls

	Endurance group (*n* = 8)	Strength group (*n* = 10)	Healthy controls (*n* = 15)	*P*-value, endurance group versus strength group	*P*-value, HIV patients versus healthy
Age (year)	53.1 (8.4)	45.9 (8.0)	47.5 (6.1)	0.09	0.5
Duration of HIV infection (year)	14 (7.4)	16 (12.2)			
Duration of antiretroviral therapy (year)	9.0 (4.6)	10.3 (3.8)			
Antiretroviral use					
NNRTI-based HAART/PI-based HHART/NNRTI-, PI-based HAART regime, *n*.	3/5/0	4/5/1			
Current NRTI use, *n*. (%)	8 (100)	10 (100)			
Lamivudine (%)	6 (75)	9 (90)			
Zidovudine (%)	4 (50)	6 (60)			
Stavudine (%)	0 (0)	1 (10)			
Tenofovir/emtricitabine (%)	3 (37.5)	1 (10)			
Abacavir (%)	3 (3.75)	4 (40)			
Current PI use, *n* (%)	5 (62.5)	6 (60)			
Current NNRTI use, *n* (%)	3 (37.5)	5 (50)			
VO_2_max (LO_2_/min)	2.5 (0.4)	2.2 (0.5)	2.5 (0.6)	0.22	0.3
Body composition					
Body mass index (kg/m2)	24.0 (3.1)	23.4 (2.5)	23.7 (1.9)	0.65	0.98
Weight (kg)	78.4 (10.0)	72.5 (9.2)	76.9 (7.9)	0.21	0.5
Trunk fat percentage (%)	70.3 (4.4)	71.5 (8.2)	56.1 (5.2)	0.71	<0.0001
Limb fat mass (kg)	3.9 (1.2)	3.4 (1.7)	6.2 (1.5)	0.45	<0.0001
Limb fat percentage (%)	26.2 (4.1)	24.8 (8.0)	40.2 (4.9)	0.66	<0.0001
Trunk-to-limb fat ratio	2.8 (0.60)	3.3 (0.61)	1.4 (0.29)	0.38	<0.001
Lean mass (kg)	59.9 (5.5)	56.2 (6.4)	58.2 (5.2)	0.18	0.9
Insulin (ρmol/liter) median (interquartile ranges)	42 (30–65)	47 (39–75)	26 (18–31)	0.29	0.0001
HOMA-IR median (interquartile ranges)	1.7 (1.4–2.6)	2.0 (1.4–2.9)	1.1 (0.76–0.3)	0.73	0.002
Insulin sensitivity					
*R*_*a*_ (μmol glucose/kg*min)					
Basal	14.3 (0.49)	14.1 (1.6)	11.8 (2.0)	0.84	0.0002
Clamp	5.9 (2.0)	6.8 (1.8)	4.0 (2.5)	0.35	0.004
Δ*R*_*a*_	8.3 (2.06)	7.3 (2.1)	7.8 (1.9)	0.34	0.9
*R*_*d*_ (μmol glucose/kg*min)					
Basal	14.3 (0.49)	14.1 (1.6)	11.8 (2.0)	0.84	0.0002
Clamp	43.0 (10.6)	38.0 (9.2)	48.6 (8.4)	0.3	0.0015
Δ*R*_*d*_	28.8 (10)	23.8 (9.4)	36.81 (7.14)	0.31	0.0015

Baseline subject characteristics are previously described in Lindegaard et al. (2008). Data are presented as mean (SD) or when indicated as median (interquartile ranges), when data were log transformed. Baseline comparisons for the HIV patients are all *P* > 0.05 by *t-*test. NNRTI, nonnucleoside reverse transcriptase inhibitor; NRTI, nucleoside reverse transcriptase inhibitor; PI, protease inhibitor; HOMA-IR, homeostatic model assessment for insulin resistance; *R*_*a*_, rate of appearance; *R*_*d*_, rate of disappearance of glucose during an euglycemic–hyperinsulinemic clamp performed in both HIV patients and healthy control; ΔR, differences between clamp and basal values.

**Table 2:** The effect of endurance and strength training on insulin sensitivity in HIV-infected patients with lipodystrophy

	Endurance				Strength			
		
	Pretraining	Posttraining	% difference	*P*-value	Pretraining	Posttraining	% difference	*P*-value
Glucose *R*_*d*_ (μmol glucose/kg body weight × min)	43.03 (10.6)	49.7 (10)	15.6	0.005	38.0 (9.2)	47.6 (15)	25	0.003
Δ*R*_*d*_	28.8 (10)	35.3 (10.5)	22.7	0.008	23.8 (9.4)	34.0 (15)	42.6	0.002

Effect of training on insulin sensitivity, as previously described in Lindegaard et al. (2008). Glucose *R*_*d*_ (glucose rate of disappearance), expressed as micromoles per kilogram body weight per minute. Data are presented as mean (SD).

### Phosphorylation of insulin signaling molecules in skeletal muscle of HIV-infected patients and controls

In response to insulin stimulation, phosphorylation of Akt^thr308^ (*P* < 0.0001), AS160^thr642^ (*P* < 0.001), and GSK3β^ser9^ (*P* < 0.05) increased in skeletal muscle of both HIV-infected patients and controls (Fig. 1). Furthermore, insulin stimulation resulted in a decreased phosphorylation of GS site 3a+b in skeletal muscle of both HIV-infected patients and controls (*P* < 0.0001). The changes (Δ) from basal to insulin-stimulated phosphorylation of Akt^thr308^ were lower in HIV-infected patients compared to controls (*P* < 0.05). There were no differences in total protein expression of Akt, AS160, GS, and GSK3 between HIV-infected patients and controls.

### Activity of GS in skeletal muscle of HIV-infected patients and controls

In response to insulin stimulation, GS activity expressed as the I form% (independent form) and FV% significantly increased in skeletal muscle of both HIV-infected patients and controls (both, *P* < 0.0001) (Fig. 2). GS activity expressed as FV% was significantly lower in skeletal muscle of HIV-infected patients (*P* < 0.01) both during basal conditions and after insulin stimulation. As expected, total GS activity was not affected by either insulin or group.

### Protein contents of Akt, AS160, HKII, GS, and GSK3 in response to endurance and strength training of HIV-infected patients

After endurance training, protein content of Akt (*P* < 0.001) and HKII (*P* < 0.05) was increased, whereas the protein content of GSK3, AS160, and GS was unaltered (Fig. 3). Strength training lead to an increased expression of HKII (*P* < 0.05), but did not affect protein level of Akt, GSK3, AS160, or GS.

### Phosphorylation of insulin signaling molecules in response to endurance and strength training of HIV-infected patients

Insulin stimulation increased phosphorylation of Akt^thr308^ in muscle biopsies obtained before and after training (*P* < 0.0001 in both training groups) (Fig. 4). Phosphorylated Akt^thr308^ was not related to total Akt, as the protein expression levels of Akt increased with endurance training (Fig. 3). Phosphorylation of AS160^thr642^ and GSK3β^ser9^ increased significantly in response to insulin in muscle biopsies obtained before and after strength training (both, *P* < 0.001), but in the group undergoing endurance training we found no statistical effect of insulin stimulation on the phosphorylation levels of AS160^thr642^ or GSK3β^ser9^. Phosphorylation of GS site 3a+b decreased significantly in response to insulin stimulation in muscle biopsies obtained before and after both endurance training (*P* < 0.01) and strength training (*P* < 0.0001). There was no effect of training at the investigated phosphorylation sites.

### Activity of GS in response to endurance and strength training of HIV-infected patients

In response to insulin stimulation, GS activity expressed as the I form% and FV% increased in muscle biopsies obtained before and after both training interventions (*P* < 0.0001, both proteins and both training groups) (Fig. 5). Their activity levels were unaffected by the exercise interventions. Total GS activity was not affected by insulin stimulation, but there was an overall effect of endurance exercise (*P* < 0.05).

## Discussion

We have previously shown that lipodystrophic patients with HIV have a markedly decreased insulin-stimulated glucose disposal compared to nonlipodystrophic HIV-negative controls, but that supervised, intensive endurance and strength training in HIV-infected patients can restore the insulin-stimulated glucose uptake (Lindegaard et al. 2008). By using muscle biopsies from the same cohort, this study further investigates the molecular mechanisms underlying the decreased insulin sensitivity in skeletal muscle of HIV-infected patients and the possible proteins involved in the beneficial effects of exercise training on insulin sensitivity. We found that lipodystrophic HIV-infected patients suffered from defects in insulin-stimulated Akt^thr308^ phosphorylation. Endurance exercise training in HIV-infected patients markedly increased basal protein expression of Akt and HKII, whereas strength training increased only basal protein expression of HKII. The findings altogether suggest that this training adaptation is associated with improved capacity for phosphorylation of glucose by HKII, rather than changes in markers of insulin signaling to glucose uptake or glycogen synthesis.

Previous studies have shown a beneficial effect of both endurance and strength training on glucose metabolism in HIV-infected patients (Jones et al. 2001; Smith et al. 2001; Yarasheski et al. 2001; Thoni et al. 2002; Driscoll et al. 2004; Dolan et al. 2006; Robinson et al. 2007), but this is the first study to investigate the molecular mechanisms involved. Thus, the strength of this study is the combination of a clinical study involving a highly specific patient group, two different supervised intensive training regimes and euglycemic–hyperinsulinemic clamps with molecular analyses in muscle biopsies.

Upon insulin stimulation of skeletal muscle, the insulin signaling pathway is activated. Akt is a critical node in the insulin signaling pathway and mediates most of the metabolic functions of insulin on skeletal muscle (Taniguchi et al. 2006). Akt is phosphorylated on threonine 308 in the activation loop by 3-phosphoinositide-dependent protein kinase 1 (PDK1) thereby enhancing the activity of the Akt kinase (Alessi et al. 1997). Our observation of a decreased insulin-stimulated phosphorylation of Akt^thr308^ in HIV-infected lipodystrophic patients compared to control subjects, indicate that the lower glucose uptake in HIV-infected patients is caused by defects involving PDK1 activation of Akt. This finding is in accordance with a study by Haugaard et al. (2005), reporting defective insulin-stimulated phosphorylation of Akt^thr308^ in skeletal muscle of lipodystrophic HIV-infected patients compared to non-lipodystrophic HIV-infected patients. Moreover, insulin-stimulated Akt^thr308^ phosphorylation is decreased in Type 2 diabetic individuals (Karlsson et al. 2005). Akt regulates glucose uptake by phosphorylating TBC1D4 (previously named AS160), a Rab GTPase-activating protein that regulates insulin-stimulated GLUT4 trafficking. The interaction of 14-3-3 regulatory proteins with TBC1D4 (AS160) phosphorylated at threonine 642 is a necessary step for insulin-stimulated GLUT4 translocation (Ramm et al. 2006). Nonetheless, we found no changes at this phosphorylation site between HIV-infected patients and controls, suggesting that potential defects in Akt signaling do not affect this mechanism. However, TBC1D4 (AS160) is phosphorylated at several sites following insulin stimulation (Sano et al. 2003), and the possibility exist that an attenuated Akt signaling transpires at other AS160 phosphorylation sites and thereby leads to attenuated GLUT4 translocation and decreased glucose uptake. Another metabolic pathway regulated by insulin via Akt is glycogen synthesis (Taniguchi et al. 2006). We performed different measures of GS activity (total activity, I-form, and FV) in the biopsies obtained before and after the insulin clamp, but none of the insulin-stimulated activities were different between HIV-infected patients and controls. In support of this finding, phosphorylation of GSK-3^ser9^ and GS at site 3a+b, which has been previously reported to be affected by HIV lipodystrophy (Haugaard et al. 2005), was comparable between groups in our study.

We observed an increased expression of Akt in skeletal muscle of HIV-infected patients following 16 weeks of endurance training. Of note, our endurance exercise protocol consisted of interval training and it is possible that steady state endurance exercise training would not have this effect on Akt expression. An increased protein expression of Akt following training has previously been observed in older subjects with and without Type 2 diabetes (Christ-Roberts et al. 2004) and in young healthy men (Frosig et al. 2007).

The increased Akt protein expression was not accompanied by changes in the phosphorylation status of Akt^thr308^. Nonetheless, an increased Akt protein expression could facilitate enhanced downstream Akt signaling reflected by changes in Akt activity without changes in phosphorylation of Akt^thr308^ (Frosig et al. 2007). Unfortunately, due to limited tissue availability, we were unable to measure Akt activity so we analyzed downstream Akt signaling. Akt signaling in skeletal muscle leads to phosphorylation of GSK3^ser9^, whereby the GSK enzyme is inactivated (Welsh and Proud 1993). This leads to dephosphorylation of GS site 3a+b, which subsequently activates the GS enzyme (McManus et al. 2005). We found no changes in the phosphorylation sites of these proteins or in the activity of GS following insulin stimulation. Thus, we have no indications that an increased Akt protein expression facilitated insulin signaling to glycogen synthesis. Neither did we observe changes in the phosphorylation state of AS160^thr642^ following the endurance training period. Thus, we did not identify a pathway whereby increased Akt protein expression would enhance insulin-stimulated glucose uptake. Interestingly, total GS activity increased with endurance training as observed both with and without insulin stimulation. This adaptation is a consistent finding following training regimes (Christ-Roberts et al. 2004; Holten et al. 2004; Frosig et al. 2007), but does not seem to result from changes in insulin signaling.

Both AMPK and PGC1a have been shown to be involved in the beneficial effects of endurance exercise on metabolism in skeletal muscle (Olesen et al. 2010; Hardie 2011). Due to limited sample availability we were unable to examine these pathways in the present study. But the possibility exist that AMPK and PGC1a contribute to the improved glucose regulation observed in the HIV patients undergoing endurance exercise.

Both endurance and strength training increased protein expression of HKII in skeletal muscle of HIV-infected patients. Increased HKII protein expression has previously been reported following endurance and strength training (Phillips et al. 1996; Frosig et al. 2007) and may be involved in the improved glucose metabolism following training. Control of glucose uptake is distributed between three steps: the rate that glucose is delivered to cells, the rate of transport into cells, and the rate that glucose is metabolized within the cells (Wasserman and Ayala 2005). An increased protein expression of HKII may facilitate faster intracellular glucose metabolism by phosphorylation of glucose to glucose-6 phosphate and thereby increase the rate of glucose metabolism within the muscle cells (Petersen and Shulman 2002; Wasserman and Ayala 2005). Indeed, overexpression of HKII in rodent skeletal muscle resulted in increased insulin-stimulated as well as exercise-stimulated glucose uptake in vivo (Halseth et al. 1999; Fueger et al. 2005). Thus, the adaptive response seen after endurance and strength training in HIV-infected patients regarding the increased protein expression of HKII may be the most important factor explaining the increased glucose uptake following training.

In conclusion, we have shown that insulin-resistant HIV-infected patients with lipodystrophy have reduced basal and insulin-stimulated GS activity (%FV) as well as impaired insulin-stimulated Akt^thr308^ phosphorylation compared to HIV-negative controls. Despite improving insulin-stimulated glucose uptake, neither endurance nor strength training changed the phosphorylation status of insulin signaling proteins or affected GS activity. However, both endurance and strength training increased protein expression HKII, thereby showing that training adaptation in HIV-infected patients is associated with improved capacity for phosphorylation of glucose by HKII, rather than changes in markers of insulin signaling to glucose uptake or glycogen synthesis.
